# Global Mapping of H3K4me1 and H3K4me3 Reveals the Chromatin State-Based Cell Type-Specific Gene Regulation in Human Treg Cells

**DOI:** 10.1371/journal.pone.0027770

**Published:** 2011-11-23

**Authors:** Yi Tian, Zhengcai Jia, Jun Wang, Zemin Huang, Jun Tang, Yanhua Zheng, Yan Tang, Qinghong Wang, Zhiqiang Tian, Di Yang, Yi Zhang, Xiaolan Fu, Jianxun Song, Shunli Liu, Jennifer C. van Velkinburgh, Yuzhang Wu, Bing Ni

**Affiliations:** 1 Institute of Immunology, PLA, Third Military Medical University, Chongqing, People′s Republic of China; 2 Beijing Genomics Institute branch in Shenzhen, Shenzhen, People′s Republic of China; 3 Department of Microbiology and Immunology, Pennsylvania State University College of Medicine, Hershey, Pennsyvania, United States of America; 4 Initiative for Collaboratory BioMedical Research, Santa Fe, New Mexico, United States of America; South Texas Veterans Health Care System, United States of America

## Abstract

Regulatory T cells (Treg) contribute to the crucial immunological processes of self-tolerance and immune homeostasis. Genomic mechanisms that regulate cell fate decisions leading to Treg or conventional T cells (Tconv) lineages and those underlying Treg function remain to be fully elucidated, especially at the histone modification level. We generated high-resolution genome-wide distribution maps of monomethylated histone H3 lysine 4 (H3K4me1) and trimethylated H3K4 (H3K4me3) in human CD4^+^CD25^+^FOXP3^+^ Tregs and CD4^+^CD25^+^FOXP3^−^ activated (a)Tconv cells by DNA sequencing-by-synthesis. 2115 H3K4me3 regions corresponded to proximal promoters; in Tregs, the genes associated with these regions included the master regulator FOXP3 and the chemokine (C-C motif) receptor 7 (CCR7). 41024 Treg-specific H3K4me1 regions were identified. The majority of the H3K4me1 regions differing between Treg and aTconv cells were located at promoter-distal sites, and *in vitro* reporter gene assays were used to evaluate and identify novel enhancer activity. We provide for the first time a comprehensive genome-wide dataset of lineage-specific H3K4me1 and H3K4me3 patterns in Treg and aTconv cells, which may control cell type-specific gene regulation. This basic principle is likely not restricted to the two closely-related T cell populations, but may apply generally to somatic cell lineages in adult organisms.

## Introduction

The CD4^+^CD25^+^FOXP3^+^ regulatory T (Treg) cells are required for proper maintenance of immunological self-tolerance and immune homeostasis [1]. Treg cells develop in the thymus as an independent CD4^+^ T cell lineage [2**]**–[****4]. It is believed that epigenetic modifications serve as an important regulatory mechanism that mediates cell fate choice between the conventional T (Tconv) cells and Tregs, but there is a paucity of information related to the epigenetic changes that occur during Treg differentiation.

Epigenetic modifications, such as methylation, acetylation and phosphorylation of histones, are implicated in regulating gene expression by controlling chromatin structure and facilitating DNA accessibility [5], [6]. In T cells, histone modifications and nucleosome positioning have been correlated with gene expression or repression [7**]**–[****11]. The functional program of Treg cells has been demonstrated to be, at least partially, controlled by miRNA epigenetic modulation [12**]**–[****14]. Moreover, constitutive gene expression of the Treg lineage-directing transcription factor (TF) forkhead box P3 (FOXP3) has been found to be dependent upon the DNA methylation status of its cell-specific enhancer [15**]**–[****18].

More than 100 differentially methylated regions (DMRs) have been identified in Treg or Tconv cell type-specific or highly expressed genes such as FOXP3, interleukin 2 receptor alpha (IL2RA), CTL-associated molecule-4 (CTLA4), CD40 ligand (CD40LG) and interferon gamma (IFNG) [19]. Unfortunately, very little information has been gleaned about the regulatory role of histone methylation during Treg lineage commitment, differentiation or cell type-specific gene regulation. Determining the global methylation profile in the distinct T cell lineages, as related to gene expression status and regulatory regions, such as promoters and enhancers, will provide significant insight into differentiation and lineage commitment processes and Treg-specific function.

General studies on histone acetylation have revealed that this particular modification is associated with the euchromatin form of DNA and active gene transcription [8], [11]. On the other hand, histone methylation has exhibited a more complex relationship with chromatin states [7], [20]. The monomethylations of one of the four core histones, H3, at lysines 27, 9 (H3K27, H3K9), H4K20, and H2BK5 are all linked to gene activation, whereas trimethylations of H3K27 and H3K9 are linked to repression [7], [20], [21]. As for H3K4, both monomethylation and trimethylation are linked to gene activation [7], [20], [21].

Acetylation has been found to be enriched in the promoter regions and at the 5′-ends of coding regions. Within the promoters, the two nucleosomes that flank the transcription start sites (TSSs) are hypoacetylated at certain lysines and are enriched in the histone H2A variant Htz1 in yeast [22**]**–[****26]. In yeast genome, the TSSs themselves are devoid of nucleosomes [27]. However, nucleosome occupancy in promoter regions (and at the TSS) is dependent on Pol II occupancy in the human genome [10], [28]. Three forms of histone methylation, monomethylated histone (H3K4me1), the dimethylated form (H3K4me2) and the trimethylated form (H3K4me3), have been characterized as strongly enriched around the TSSs, whereas H3K36me3 peaks near the 3′-ends of genes [29**]**–[****31].

The chromatin immunoprecipitation-sequencing (ChIP-Seq) technique developed in recent years combines the use of modification-specific antibodies for ChIP with next-generation high-throughput sequencing-by-synthesis, and has revolutionized our ability to monitor the global incidence of histone modifications. ChIP-Seq profiles for protein–DNA association have been successfully used to identify distal and proximal regulatory elements with high spatial resolution [7]. In this study, we aimed to take advantage of the fine resolution afforded by the ChIP-Seq assay to generate, for the first time, genome-wide distribution profiles of H3K4me1 and H3K4me3 in human Treg cells and activated (a)Tconv cells.

Previous ChIP analysis followed by microarray sequencing-by-hybridization of the 1% of the human genome represented by the ENCODE regions indicated that H3K4me1, but not H3K4me3, was enriched around distal *cis*-elements for the E1A binding protein p300 (EP300), while both modifications were enriched at promoters [32]. Furthermore, the chromatin state at promoters was found to be largely invariant across diverse cell types. In contrast, the enhancers identified in different cell types appeared to have cell type-dependent chromatin modification patterns [33], and the cell type-specific presence of chromatin marks at enhancers, such as of H3K4me1, was closely correlated with cell type-specific expression of the putative gene targets of these enhancers [34]. Thus, enhancers may be more dynamically regulated in different cell types and are likely principal mediators of cell type-specific gene expression. Using the global profile of methylation distribution in Tregs and aTconv cells, we also aimed to discover novel enhancer regions that mediated differential gene expression.

Here we present the comprehensive genome-wide dataset of lineage-specific H3K4me1 and H3K4me3 patterns in Treg and aTconv cells. The majority of the H3K4me1 regions found to differ between Treg and aTconv cells were located at promoter-distal sites. I*n vitro* reporter gene assays were used to evaluate and identify novel enhancer activity. These global methylation profiles represent a crucial foundation from which future studies will elucidate the genetic mechanisms that regulate differentiation decisions, lineage commitment and gene regulation in Tregs.

## Materials and Methods

### Cell purification and culture

Mononuclear cells (MNCs) were isolated from leukapheresis products of healthy volunteers by density gradient centrifugation over Ficoll-Hypaque solution (Biochrome AG, Germany). CD4^+^CD25^+^ T cells were enriched using the human CD4^+^CD25^+^ Regulatory T Cell Isolation Kit and the Midi-MACS separation system (both by Miltenyi Biotec, Germany). The isolated CD4^+^CD25^+^ T cells were then stained with CD4-FITC and CD25-PE (both from BD Biosciences, USA), and their purity was detected with a FACS-Aria high-speed cell sorter (BD Biosciences). The purity of cells after sortings was determined to reach above 98%.

MACS-purified CD4^+^CD25^+^ regulatory T cell populations were monoclonally expanded *in vitro* over a period of eight to nine weeks using the Dynabeads® Human Treg Expander (Invitrogen, USA). Briefly, isolated cells were stimulated with magnetic polysterene beads coated with a mixture of monoclonal antibodies against CD3 and CD28 in the presence of high-dose recombinant human IL-2 (rhIL-2, 300 U/mL: Proleukin; Chiron, USA), as described in the manufacturer's instructions. The expanded cells were then stained with cell surface anti-CD4-FITC, anti-CD25-PE antibodies and intracellular anti-FOXP3-APC antibody (eBioscience, USA), and the fixed cells were separated by fluorescence activated cell sorting (FACS) into batches of CD4^+^CD25^+^FOXP3^−^ activated Tconv (aTconv) and CD4^+^CD25^+^FOXP3^+^ regulatory T cells.

Jurkat cells (human T cell leukemia) were grown in 90% 1640-RPMI (PAN Biotech GmbH, Germany) plus 10% fetal bovine serum (FBS) supplemented with 2 mM L-glutamine (Biochrome, Germany), MEM non-essential amino acids, sodium pyruvate, MEM vitamins, 50 U/mL penicillin/streptomycin, and 50 nM beta-mercaptoethanol (all from Gibco, USA) in a humidified atmosphere at 37°C and 5% CO_2_.

Written, informed consent was obtained from all subjects prior to participation, and this study was approved by the ethics committee of the Third Military Medical University, Chongqing, China.

### Suppression assay

CD4^+^CD25^-^ T cells selected from PBMCs with anti-CD4 MACS-beads were labeled with 2 µM of the intracellular fluorescent dye 5-(and -6)-carboxyfluorescein diacetate succinimidyl ester (CFSE; Invitrogen) for 10 minutes at 37°C, and washed twice with PBS. Aliquots of 2×10^4^ sorted CD4^+^CD25^−^ T cells were seeded in wells on a 96-well U-bottom plate pre-coated with anti-CD3 (2 µg/mL; BD Biosciences) and co-stimulated with either soluble anti-CD28 (1 µg/ml; BD Biosciences) alone or in the presence of expanded CD4^+^CD25^+^ T cells at different ratios, as indicated. Co-cultures were harvested after four to five days of incubation and analyzed on a FACS Calibur flow cytometer.

### ChIP and ChIP-Seq

The procedure of ChIP-Seq was carried out as previously described [7]. The Treg and aTconv cells were used for ChIP analysis. To map enzyme target sites, 2×10^6^ cells were crosslinked with formaldehyde and sonicated to obtain chromatin fragments of 200 to 300 bp. Sonicated chromatin was pre-cleared and incubated with 2 µg of anti-H3K4me1 (Abcam, United Kingdom), anti-H3K4me3 (Abcam) or anti-rabbit IgG (Upstate, USA) overnight at 4°C. The crosslinks were reversed, and DNA was treated sequentially with Proteinase K and RNase A, and purified using the Qiaquick PCR-purification kit (Qiagen, Germany). ChIP samples were tested for enrichment by qPCR. For ChIP-Seq, the precipitated DNA was repaired using PNK and Klenow enzyme, and ligated to adapters according to manufacturer's instructions. Subsequently, PCR-amplified fragments of approximately 220 bp were sequenced using the Solexa 1 G Genome Analyzer following manufacturer's protocols (www.illumina.com). The ChIP-Seq data have been now accessible in NCBI's Gene Expression Omnibus (GSE26427).

### ChIP-seq reads mapping to genomic regions

ChIP-seq reads of ∼35 bp were mapped to the University of California, Santa Cruz (UCSC) human genome (hg18) by SOAP, which allowed a uniquely aligned read to have up to two mismatching bases [35], [36]. The output of the SOAP analysis data was converted to browser-extensible data (BED) files in order to view the data in the UCSC Genome Browser [37].

### Identification of H3K4me1 and H3K4me3 peaks

The uniquely aligned reads by SOAP were considered in peaks calling. To eliminate noise and account for unequal total numbers, we used a defined analysis model (Model-based Analysis of ChIP-Seq, MACS) with default parameters to find peaks , which were called “peaks” of H3K4me1 and H3K4me3 [38]. The results include peak location, peak sequence, etc.

### Distribution of H3K4me1 and H3K4me3 peaks

The overall profile of the H3K4me1 and H3K4me3 distribution was generated by dividing the human genome into four regions [39]: proximal promoters (1 kb upstream and downstream of the transcription start site (txStart), based on annotated “known genes” from the UCSC Genome Browser [37]); exons; introns; and intergenic sequences.

### Identification of common and lineage-specific H3K4me1 and H3K4me3 peaks

We compared the location of each peak in Treg and aTconv cells for H3K4me1 and H3K4me3. For identification of common peaks, the location of peaks has to be overlapped in both lineages with a minimal distance of 1 bp. Furthermore, the lineage-specific peaks were defined as peaks in one lineage that did not overlap with any other peaks in the other lineage.

### Identification of common and lineage-specific proximal promoters or genes

Proximal promoters enriched by H3K4me3 in the two lineages were compared each other to determine the common and cell-type specific proximal promoters. As a single proximal promoter is usually associated with one or more genes, we compared the gene(s) associated with each of the proximal promoters in each cell type to determine the common and cell-type specific genes.

### Profiles of the tag density of modifications

For each gene, uniquely mapped tags (reads) were summed in 125bp windows (40 windows per region) for the regions ranging from 5 kb upstream of txStart to the txStart itself and from the transcription end site (txEnd) to 5 kb downstream of the txEnd, respectively. Within the gene body, every gene was splitted into 40 windows. All window tag counts were normalized by the total number of bases encompassed within the windows and the total read number from sequencing of the given library [11].

### Quantitative real-time PCR

Total RNA was extracted from the expanded and sorted Treg and aTconv cells by Trizol Reagent (Invitrogen, USA). The quantity of total RNA was measured by a NanoDrop spectrophotometer (Agilent Technologies, USA), and 500 ng was used to synthesize cDNA with a Reverse Transcription Kit (TaKaRa, Japan). GAPDH was used as the endogenous control. PCR was carried out in a 25 µl reaction with 0.5 mM gene-specific primers (Table S1) and using a SYBR Green Kit (TaKaRa) for 40 cycles in a Rotor-Gene 6000 (Gene Company Limited, Australia). The 2^−ΔΔ^ CT method was used to calculate expression relative to the GAPDH housekeeping control [40].

### Reporter assays

The selected H3K4me1 and H3K4me3 enriched regions (500–1000 bp) were PCR-amplified from human genomic DNA and cloned directly into the pGL3-promoter vector (Promega, USA). Primer sequences are listed in Table S2. All inserts were verified by sequencing. One-million Jurkat cells were co-transfected using DEAE-dextran, with 1.0 mg of each reporter plasmid and 0.15 mg of a Renilla control vector (Promega). After transfection, cells were left untreated or stimulated with 20 ng/mL PMA and 1 mM ionomycin (1 mg/mL). Triplicate transfections were harvested after 24 h of incubation. Cell lysates were assayed for firefly and Renilla luciferase activities using the Dual-Luciferase Reporter Assay System (Promega) on a Lumat LB9501 luminometer (Berthold, Germany). Firefly luciferase activity of individual transfections was normalized against the Renilla luciferase activity.

### Statistical analysis

The two-tailed Student's *t-*test was used in the analysis of mRNA expression and of luciferase activity. Significance was defined by a *P*-value <0.05.

## Results

### Expansion and purification of human CD4^+^CD25^+^FOXP3^+^ Treg cells

**Figure 1:**
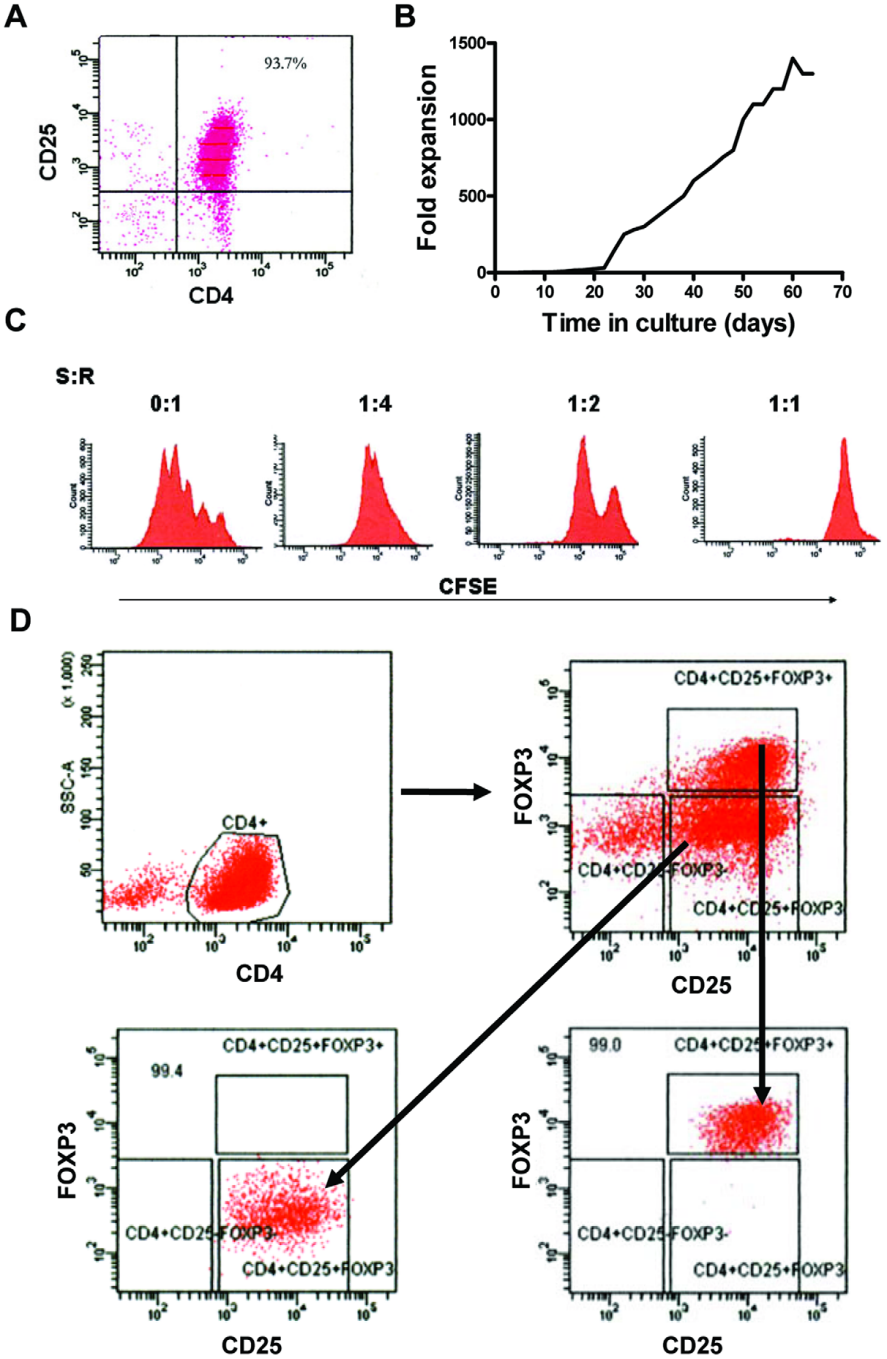
Expansion and purification of human CD4^+^CD25^+^FOXP3^+^ Treg cells. **Panel A**, Isolation and purity (% indicated) of CD4^+^CD25^+^ T cells from human PBMCs. **Panel B**, Expansion rates of sorted CD4^+^CD25^+^ T cells *in vitro.* **Panel C**, Evaluation of the suppressive capacity of expanded cells. **Panel D**, FACS-sorting of CD4^+^CD25^+^FOXP3^+^ regulatory T cells and CD4^+^CD25^+^FOXP3^−^ T cells after expansion. The purity of each sub-population is indicated. S:R, Suppressor:Responder ratios

Due to the low frequency of Treg cells present in peripheral blood, we expanded the Treg cell population *in vitro* to obtain enough cells for analysis. First, we purified CD4^+^CD25^+^ T cells from PBMCs of healthy human volunteer subjects by using the Magnetic-activated cell-sorting method (MACS). The purity of products was determined to be >93% (Fig. 1A). Then, the CD4^+^CD25^+^ T cells were exposed to artificial antigen-presenting cells for repeated stimulation via CD3 and CD28 in the presence of high-dose IL-2, which resulted in profound monoclonal proliferation of up to 1000-fold expansion within an eight to nine week span (Fig. 1B). We then tested the suppressive activity of the expanded CD4^+^CD25^+^ T cells by evaluating their ability to inhibit the proliferation of autologous CD4^+^CD25^−^ T cells after allogeneic stimulation. Results from the mixed lymphocyte reaction (MLR) assay showed that the proliferation of CD4^+^CD25^−^ responder T cells was inhibited, in a dose-dependent manner, by the expanded CD4^+^CD25^+^ T cells (Fig. 1C). Because CD25 is known to be expressed on activated T cells derived from CD4^+^CD25^−^FOXP3^−^ T cells, the expanded CD4^+^CD25^+^ T cells were expected to include FOXP3^−^ T cells. To obtain high-purity of CD4^+^CD25^+^FOXP3^+^ Treg cells, which represent the intrinsic Treg cells, we purified the triple-positive T cells from the expanded CD4^+^CD25^+^ T cells by the FACS method, and the purity of CD4^+^CD25^+^FOXP3^+^ Treg and activated conventional CD4^+^CD25^+^FOXP3^−^ T cells (aTconv) reached 99.0% and 99.4%, respectively (Fig. 1D).

### Direct sequencing analysis of ChIP DNA samples

We used the high-throughput ChIP-Seq approach to generate genome-wide H3K4me1 and H3K4me3 maps of human CD4^+^CD25^+^FOXP3^+^ Treg cells and CD4^+^CD25^+^FOXP3^−^ aTconv cells. The sequencing procedure required a one-step adaptor ligation and limited PCR amplification (18 cycles) of ChIP DNA molecules, followed by cluster generation and sequencing-by-synthesis. The read/peak numbers for each library in each cell type were shown in Table S3.

Prior to and post sequencing on the Solexa 1 G Genome Analyzer, the ChIP samples were confirmed for the target sites in both cell types by regular ChIP-qPCR with the indicated primers (Table S4 and S5) [41]. The qPCR results were highly consistent with ChIP-Seq data as expected (Fig. S1 and S2).

### Genome-wide maps of H3K4me3 modifications in human CD4^+^CD25^+^ T cell lineages with or without FOXP3 expression

**Figure 2:**
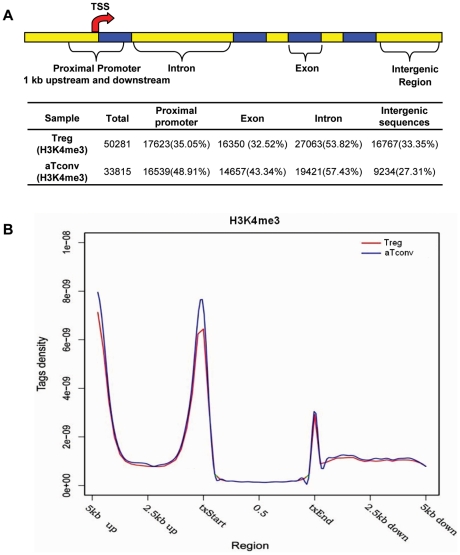
Distribution and tag density of H3K4me3 enriched peaks. Panel A , An illustration of the four regions that the human genome was divided into: proximal promoters, exons, introns, and intergenic sequences [39]. The number of islands detected for each sample is listed (Total), followed by the islands among genomic regions with the percentage they comprise listed in the parenthesis. **Panel B**, Tag density of H3K4me3 enriched peaks in Treg and aTconv cells. Tag density was plotted by splitting upstream (5Kb), downstream(5Kb) and gene body into 40 windows, respectively. Tag density = number of reads in specific region/(reads total number×specific region length).

To obtain an overall picture of the H3K4me3 distribution, we divided the entire human genome into four distinct regions, according to the annotated “known genes” from the UCSC Genome Browser [37], [39]: proximal promoters (1 kb upstream and downstream of the TSSs), exons, introns, and intergenic sequences. Results showed that about 35% and 49% of H3K4me3 islands were located in proximal promoter regions for Treg cells and aTconv cells, respectively (Fig. 2A). Examination of those H3K4me3 tags (reads) located within gene bodies, and their 5′- and 3′-end 5 kb extended regions, also revealed enrichment of H3K4me3 islands near TSSs (Fig. 2B). These results are consistent with recent observations from others that have indicated that H3K4me3 associates extensively with proximal promoters of active genes in human T cells, as well as in human and murine embryonic stem cells [7], [20], [42]–[44].

**Figure 3:**
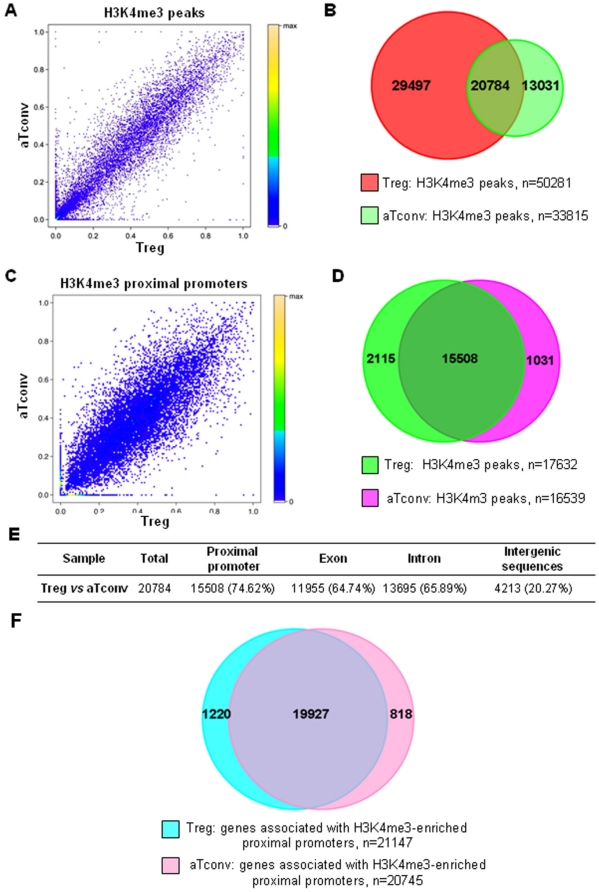
Comparison of H3K4me3 enriched regions, proximal promoters or related genes between Treg and aTconv cells. Panel A , Correlation of H3K4me3 enriched regions between Treg and aTconv cells. Each spot represents a peak. The X- and Y-axis represent the ratio of the total reads of the H3K4me3 enriched peak to the length of the proximal promoter of this gene in Treg or aTconv cells, respectively. **Panel B**, Overlapping H3K4me3 enriched regions between Treg and aTconv cells. **Panel C**, Correlation of proximal promoters marked by H3K4me3 between Treg and aTconv cells. Each spot represents an individual peak. The X- and Y-axes are the same as for Panel A. **Panel D**, The high degree of overlapping proximal promoters marked by H3K4me3 between Treg and aTconv cells. **Panel E**, Summary of the distribution of overlapping regions enriched by H3K4me3 in Treg and aTconv cells. **Panel F**, Venn diagram showing the difference of genes associated with H3K4me3 enriched proximal promoters between Treg and aTconv cells.

We compared the H3K4me3 enriched regions between Treg and aTconv cells, and found that the coefficient correlation was 0.92 and that there were 20784 H3K4me3 islands that overlapped in the two cell types (Fig. 3A and B). Furthermore, about 75% of these overlapping islands were located in proximal promoters (Fig. 3E). We then compared the H3K4me3 enriched proximal promoters of Treg and aTconv cells, and determined that the coefficient correlation was 0.83 and that there were 15508 overlapping H3K4me3 enriched proximal promoters (Fig. 3C and D). In addition to these overlapping islands we also found that nearly 30000 H3K4me3 islands were Treg cell-type specific, and about 7% of those were associated with proximal promoters (Fig. 3B and D). We also analyzed the particular genes related to the H3K4me3 enriched proximal promoters, and found that most of the genes were common between the two cell types; only 1220 related genes were Treg cell-type specific (Fig. 3F). These results suggested that most of the genes were expressed in both Treg cells and aTconv cells, and the distinct properties of development and function of Treg cells might, in fact, be due to the unique H3K4me3 modification of Treg cell type-specific genes like FOXP3.

### Disparate H3K4me3 modification of signature genes between Treg and aTconv cells

**Figure 4:**
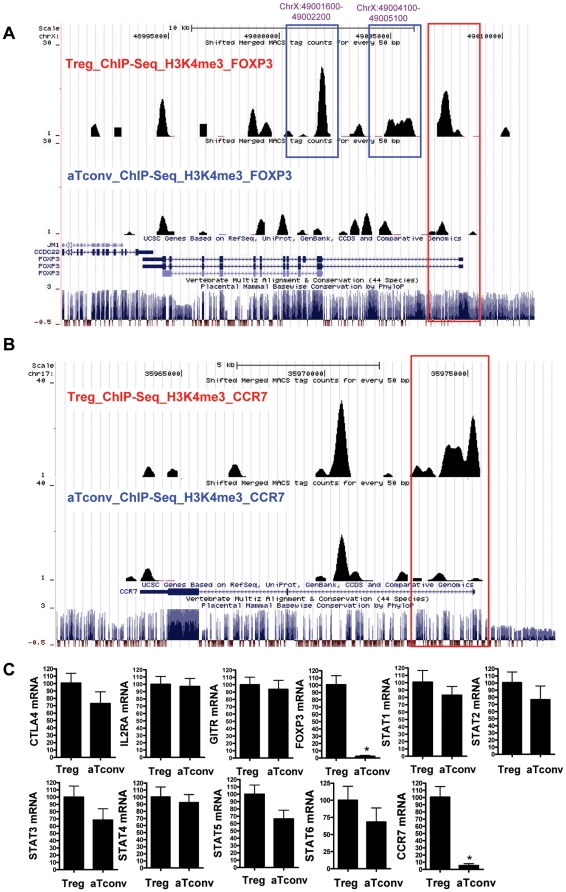
H3K4me3 modifications of cell signature genes and common genes in Treg and aTconv cells, as analyzed by the UCSC hg18 Genome Browser. The following tracks are shown (from top to bottom): Window position; ChIP-Seq tag counts for H3K4me3 modifications in Treg cells; ChIP-seq tag counts for H3K4me3 modifications in aTconv cells; UCSC genes based on RefSeq; mammalian consensus. The red frames indicate the H3K4me3 modifications in proximal promoters. Panel A, H3K4me3 modification on the FOXP3 loci (ChrX:48990000–49010000). The two intragenic H3K4me3 islands, located about 6 kb (ChrX:49001600–49002200) and 4 kb (ChrX:49004100–49005100) downstream of the FOXP3 promoter in Treg cells, are framed in blue. Panel B, H3K4me3 modification on the CCR7 loci (Chr17:35960621–35978190). Panel C, Expression of cell signature genes and common genes. The mRNA expression levels of signature genes and common genes are shown for Treg and aTconv cells, as measured by real-time PCR assays. The 2^−ΔΔ^ CT method is used to quantify the expression relative to the GAPDH housekeeping control. Data are shown as fold-induction relative to Treg cells and are the mean ± standard error of the mean (SEM) from three independent experiments. *, *P*<0.01, *vs* Treg cells.

Because the T cell subsets represented distinct and stable cell lineages, we inferred that the signature genes corresponding to their respective phenotypes would harbor unique H3K4me3 marks in their proximal promoters, consistent with the corresponding gene expressions in that particular lineage. We first examined the H3K4me3 pattern for IL2RA, CTLA4, TNFRSF18 and FOXP3 genes, each of which encodes the defining lineage markers for Treg cells. Results showed that IL2RA, CTLA4 and TNFRSF18 genes were marked in their promoters by H3K4me3 in both Treg and aTconv cells; this finding was consistent with their respective expression levels detected in activated T cells derived from CD4^+^CD25^−^ T cells (Fig. S3A-C). In contrast, FOXP3, a gene that is required for Treg cell development and functions, was marked in its proximal promoter by H3K4me3 in Treg cells, but not in aTconv cells (Fig. 4A). We detected a 50-fold increase in the expression level of FOXP3 mRNA in Treg cells, as compared to aTconv cells; comparable expression levels of IL2RA, CTLA4 and TNFRSF18 mRNA were observed between the two cell types (Fig. 4C).

We also examined the mRNA expression levels of other genes that were found to be marked in their proximal promoters by H3K4me3 in Treg and/or aTconv cells, such as the STATs and CCR7. The mRNA expression levels of these genes were consistent with the H3K4me3 status observed for their proximal promoter. For example, STAT family TFs are crucial for proper T cell differentiation; however, their expression is not sufficient to drive lineage commitment [39]. Consistent with their ubiquitous expression patterns, we found that most STATs were marked in their promoter regions by H3K4me3, in both the Treg and aTconv cells (Fig. S3D-I). Real-time PCR assays showed that the mRNA expression levels of all the STATs were comparable among the two lineages (Fig. 4C). In contrast, the promoter for the CCR7 gene was marked by H3K4me3 only in Treg cells (Fig. 4B), and the real-time PCR assay showed an approximate 20-fold increase in its expression as compared to that in aTconv cells (Fig. 4C). Based on the above results, we predict that Treg differentiation and lineage commitment are associated with specific H3K4me3 events in the 1220 cell-type specific genes (Fig. 3F) that were marked in their proximal promoters by H3K4me3 only in Treg cells and not in aTconv cells.

Apart from the H3K4me3 islands in promoters, there were about 60% H3K4me3 islands located in non-promoter regions, a finding which may be indicative of enhancers [7], [11]. Two regions of particular interest were the intragenic H3K4me3 islands located about 6 kb (ChrX:49001600-49002200) and 4 kb (ChrX:49004100-49005100) downstream of the FOXP3 promoter in Treg cells (Fig. 4A). By using online tool “TFSEARCH: Searching Transcription Factor Binding Sites (ver 1.3)”[45], we found both islands contained multiple TF target sites, including those for p300, AML1 and STATs. As such, this region may serve as an enhancer to regulate the transcription of the FOXP3 gene in Treg cells.

### Genome-wide maps of H3K4me1 modifications in human CD4^+^CD25^+^ T cell lineages with or without FOXP3 expression

**Figure 5:**
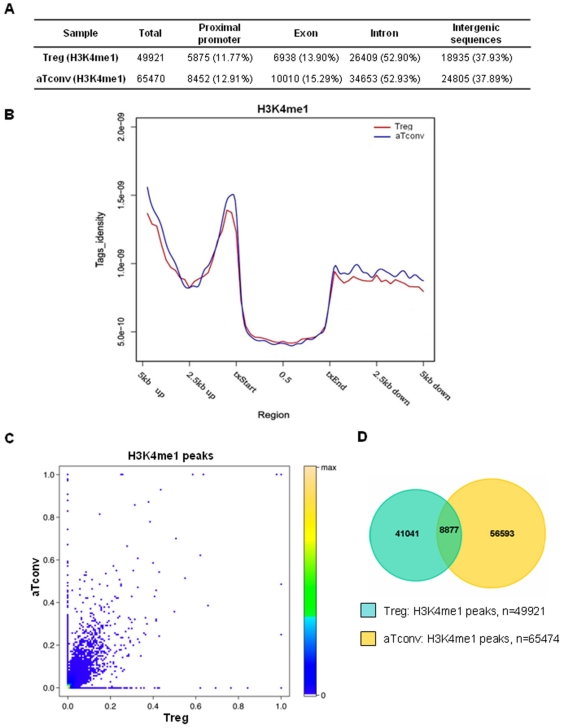
Genome-wide distribution and comparison of H3K4me1 modifications between Treg and aTconv cells. Panels A and B , Distribution and tag density of H3K4me1 enriched peaks. **(A)** Distribution of H3K4me1 enriched peaks. The number of islands for each sample is listed as the total identified islands (Total), followed by the islands among genomic regions with the corresponding percentage listed in the parenthesis. **(B)** For each gene, uniquely mapped tags were summed in 125 bp windows from 5 kb upstream of the txStart to the txStart, and from the txEnd to 5 kb downstream. **Panels C and D**, Comparison of H3K4me1 enriched regions between Treg and aTconv cells. **(C)** Correlation of H3K4me1 enriched regions between Treg and aTconv cells. Each spot represents a single peak. The X- and Y-axis represent the ratio of the total reads of H3K4me1 enriched peak to the length of this gene in Treg or aTconv cells, respectively. **(D)** Overlapping H3K4me1 enriched regions between Treg and aTconv cells.

Previous studies have suggested that H3K4me1 at promoter-distal sites is often associated with the presence of an enhancer [7], [11], [32]. We, thus, generated genome-wide H3K4me1 maps in human Treg and aTconv cells to compare the predicted enhancers in both cell types. Results showed that more than half of the total identified H3K4me1 islands were located in introns in both aTconv and Treg cells (Fig. 5A). Interestingly, examination of those H3K4me1 tags found within gene bodies and their 5′- and 3′-end 5 kb extended regions also revealed that the H3K4me1 enrichment status of proximal promoters was higher than those in other regions (Fig. 5B).

When comparing the H3K4me1 enriched regions in Treg and aTconv cells, we found that the coefficient correlation was only 0.48 and there were only 8897 overlapping H3K4me1 islands present among the 115391 total regions between both cell types (Fig. 5C and D). These results indicated that most of the H3K4me1 islands were cell-type specific. More importantly, they suggested that enhancers represent the most variable class of transcriptional regulatory element between Treg and aTconv cells, and were probably primary mediators of Treg cell-type specific patterns of gene expression.

**H3K4me1 modifications of cell signature genes and verification of enhancer activity**

**Figure 6:**
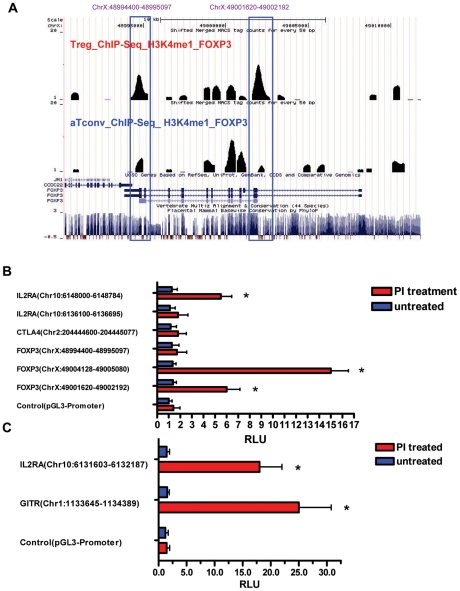
Prediction of enhancers and verification of activity for cell signature genes. Panel A , UCSC hg18 genome browser views of H3K4me1 modifications of the cell signature gene FOXP3 loci (ChrX:48990000–49010000) in Treg and aTconv cells. The following tracks are shown (from top to bottom): genes' location; ChIP-Seq tag counts for H3K4me1 modifications in Treg cells; ChIP-Seq tag counts for H3K4me1 modifications in aTconv cells; UCSC genes based on RefSeq; mammalian consensus. The blue frames indicate the Treg cell-type specific H3K4me1 enriched regions. **Panels B** Functional analysis of enhancer candidates specific for Treg cells. These fragments are all enriched by H3K4me1 modifications except the FOXP3 (ChrX:49004128–49005080) fragment, which is H3K4me3 modified and identified as an enhancer in the published literatures. Panel C, Functional analysis of the enhancer candidates common in both Treg and aTconv cells, which are all H3K4me1 modified. All the selected enhancer candidates were cloned into the luciferase vector pGL3-Promoter, respectively. The indicated plasmids were transiently transfected into Jurkat T cells that were stimulated with or without PMA and ionomycin (PI) after transfection. Luciferase activity was normalized against the activity of a co-transfected Renilla construct. Mean values ± SEM are shown relative to the pGL3-promoter alone. * indicates a statistically significant difference between the cloned *vs* control plasmids (*P*<0.01; paired Student's *t*-test).

Among the 18081 total genes that were H3K4me1 modified in Treg cells (Fig. 5A), we selected a subset of the cell signature genes to further examine the H3K4me1 patterns and verify the activities of enhancers predicted to be related to these genes. The signature gene subset included IL2RA, CTLA4, TNFRSF18 and FOXP3 genes, which are known to be highly or specifically expressed in Treg cells. We identified some Treg cell-specific H3K4me1 regions, including: a region in intron 1 of FOXP3 that was also enriched by H3K4me3 (Fig. 4A and 6A) [ChrX:49001620–49002192]; a region in the last exon of FOXP3 (Fig. 6A) [ChrX:48994400–48995097]; a region in intron 2 of the CTLA4 (Fig. S3J) [Chr2:204444600–204445077]; a region in intron 1 of IL2RA [Chr10:6136100–6136695] and a region upstream of IL2RA (Fig. S3K) [Chr10:6148000–6148784]. We were unable to identify any Treg cell-specific H3K4me1 regions for the TNFRSF18 gene (Fig. S3L).

Previous studies have suggested that H3K4me1 at promoter-distal sites are often associated with enhancer function. A general property of such enhancers is the ability to increase transcriptional activity in a heterologous context. As this type of function can be readily studied using traditional reporter gene assays, we selected the five Treg cell-specific H3K4me1 regions described above to evaluate their heterologous enhancer activities. As shown in Figure 6B, only two of the five regions examined showed enhancer activity. Interestingly, the majority of regions that did not show enhancer activity in Jurkat cells corresponded to Treg cell-specific H3K4me1 enriched regions. In line with this finding, a H3K4me1 region in intron 1 of the IL2RA gene (Fig. S3K) [Chr10:6131603–6132187] and a H3K4me1 region upstream of the TNFRSF18 gene (Fig. S3L) [Chr1:1133645–1134389], which were enriched in both Treg and aTconv cells, did exhibit enhancer activity in Jurkat cells (Fig. 6C). Since Jurkat T cells represent a leukemic counterpart of conventional T cells, it is very possible that they lack Treg cell-specific TFs that are necessary for enhancer functions of these particular regions. However, some Treg cell-specific H3K4me1 regions did function even in Jurkat cells, suggesting that the relevant TFs required for enhancer activity at these sites were, at least, available.

### Comparison of H3K4me3 and H3K4me1 enriched regions in Treg or aTconv cells

**Figure 7:**
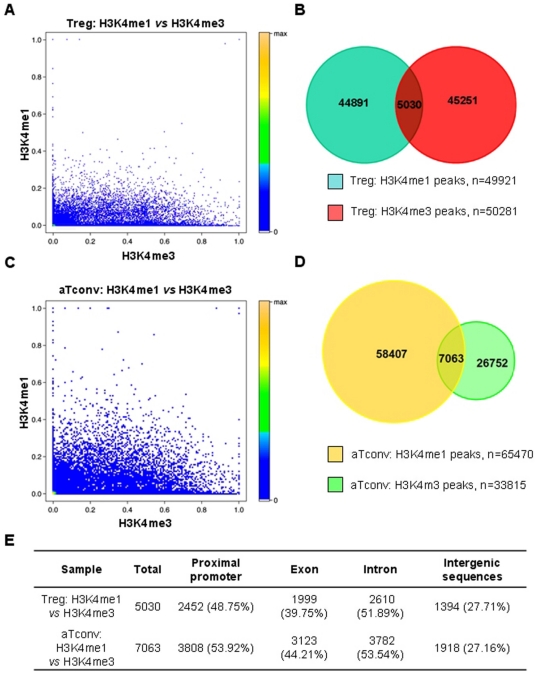
Comparison of H3K4me1 and H3K4me3 enriched regions in Treg or aTconv cells. Panel A , Correlation of H3K4me1 and H3K4me3 enriched regions in Treg cells. Each spot represents a single peak. The X-axis represents the ratio of total reads of the H3K4me3 enriched peak to the length of corresponding gene, while the Y axis represents the ratio of the total reads of the H3K4me1 enriched peak to the length of corresponding gene. **Panel B**, Regions enriched by H3K4me1 and H3K4me3 in Treg cells. **Panel C**, Correlation of H3K4me1 and H3K4me3 enriched regions in aTconv cells. Each spot represents a single peak. The X- and Y-axes are the same as for Panel A. **Panel D**, Regions enriched by H3K4me1 and H3K4me3 in aTconv cells. **Panel E**, Summary of the distribution of the regions enriched by H3K4me1 and H3K4me3 in Treg or aTconv cells.

We also compared the H3K4me1 and H3K4me3 enriched regions in the same sample, and determined that the coefficient correlation was only 0.16 in Treg cells and 0.19 in aTconv cells. Furthermore, there were only 5030 overlapping H3K4me1 regions and 7063 overlapping H3K4me3 regions (Fig. 7). These results indicated that H3K4me1 modified regions with potential regulatory function were seldom overlapped with H3K4me3 modified regions in the whole genome of human Treg and aTconv cells.

## Discussion

In this study, we obtained high-purity of human CD4^+^CD25^+^FOXP3^+^ Treg cells and aTconv cells by combining *in vitro* expansion, MACS- and FACS- sorting methods (Fig. 1). As reported by other researchers employing this technique, these cells maintained all phenotypic, functional and epigenetic Treg cell characteristics, even after extensive *in vitro* expansion [46], [47]. We utilized these cells for ChIP-Seq analysis to generate high-resolution maps of the genome-wide distribution of H3K4me1 and H3K4me3 in both cell subtypes. Ultimately, we identified a number of cell type-specific H3K4me1 regions and H3K4me3 marked proximal promoters in Treg cells. The majority of the differential H3K4me1 regions were found to be located in promoter-distal sites, and we selected some for verification of their enhancer activity by using reporter gene assays.

CD4-positivity and CD25-positivity have long been considered as the cell-specific indicators of Treg cells. However, CD4^+^CD25^-^ T cells were demonstrated to be able to up-regulate their CD25 expression upon activation by antigen, indicating that CD4 and CD25 double-positive T cells actually represent a heterogeneous cell population and these surface markers are not sufficient identifiers of Treg cells. Thus, we used CD4^+^CD25^+^FOXP3^+^ triple expression to define Treg cells since FOXP3 gene expression is essential for Treg cell function. We carried out comparative analysis of the genome wide epigenetic methylation status for H3K4 in CD4^+^CD25^+^FOXP3^+^ (Treg) and CD4^+^CD25^+^FOXP3^-^ T cells (aTconv).

The low frequency of Treg cells in normal human peripheral blood has thus far limited the detailed characterization and potential clinical application of human Treg cells. In many previous studies, Treg expansion has been carried out to obtain enough cell material to perform analysis. Unfortunately, CD4 and CD25 were usually used to identify the Treg cells. Here, we found that although CD4^+^CD25^+^ T cells were expanded up to 1000-fold, most of the expanded cells were FOXP3-negative. Thus, we performed FACS-sorting to obtain high-purity Treg cells with CD4, CD25 and FOXP3 expressions immediately prior to our ChIP-Seq assay.

Previously, Heintzman determined the chromatin modification states at high resolution along 30 Mb of the human genome, and found that active promoters were marked by H3K4me3 and enhancers by H3K4me1 [32]. We also found that most proximal promoters enriched by H3K4me3 were common between the Treg and aTconv cells, suggesting that the related genes of the proximal promoters were co-expressed in the two lineages. Although some genes are widely used as markers for Treg cells, such as IL2RA, CTLA4 and TNFRSF18, accumulating evidence has unfortunately suggested that these markers are not strictly Treg-specific. Upon activation, all T cells express CD25, the alpha-chain of the IL-2 receptor [48], [49], and its combination with IL-2 is essential for T cell clonal expansion. CTLA-4, which is the receptor for APC-B7, negatively regulates the IL-2 production of the newly activated T cell and inhibits further T cell proliferation upon binding of B7 and is up-regulated on all CD4^+^ and CD8^+^ T cells, two to three days following activation [49], [50]. Similarly, the expression of TNFRSF18, which is a possible target molecule in cell contact-dependent suppression, is induced in T cells upon activation [49], [51]. This could explain why we observed H3K4me3 in the proximal promoters of these genes in aTconv cells. STAT family TFs are critical for T cell differentiation; however, their expression is not sufficient to drive lineage commitment. Consistent with the ubiquitous expression patterns of STAT family TFs, we found that most STATs were marked in their promoter regions by H3K4me3, in both Treg and aTconv cells. Based on these results, we predicted that the common 19927 genes between Treg and aTconv cells (Fig. 3F) may be expressed in both lineages.

We found that, apart from the common H3K4me3 promoters, there were also some Treg cell-type specific proximal promoters marked by H3K4me3, such as FOXP3 (Fig. 4A and C). It may be these types of specific proximal promoters, especially the FOXP3, that are responsible for the differences between Treg cell and aTconv cells. The proximal promoter of FOXP3, which is believed to serve as a master regulator of Treg cells, was found to be enriched by H3K4me3 in Treg cells. Moreover, a 50-fold higher mRNA expression level was observed in Treg cells, as compared to aTconv cells. This nearly exclusive expression of FOXP3 in Treg cells was in accordance with the current concept that FOXP3 represents the critical TF of Treg cells. In addition, we also found that the CCR7 gene was marked by H3K4me3 in its proximal promoter only in Treg cells, and exhibited a nearly 20-fold increase of mRNA expression in Treg, as compared to aTconv cells.

H3K4me3 is usually associated with promoters, and its occurrence at enhancers remains a topic of debate. Whereas Heintzman, *et al*. found little or no H3K4me3 at p300-associated enhancers [32], Barski, *et al*. identified all three methylation states at the related functional enhancers [7]. It is, therefore, unclear whether the promoter-distal H3K4me3 sites identified in this study are associated with uncharacterized functional transcription units, or whether they are able to act as enhancer regions themselves. For example, we found there was a region located about 6 kb downstream of the FOXP3 promoter (ChrX:49001620–49002192), which showed enhancer activity in transient transfection assays; the existence of this region suggests that there may be some non-promoter H3K4me3 regions associated with enhancers. Another region located about 4 kb downstream of the FOXP3 promoter was specifically enriched by H3K4me3 in Treg cells (ChrX:49004128–49005080), and also exhibited enhancer activity; interestingly, previous studies have shown that this region was enriched for STAT5 consensus sites. Treg cell survival critically depends on interaction with IL-2. The TF STAT5 is activated through the IL-2 receptor [52], has an essential role in Treg cell homeostasis [53], and is known to regulate the lineage-specific TF FOXP3 through an intronic, methylation-sensitive enhancer [54]. Together, all the data indicate that certain promoter-distal H3K4me3 modified regions may have enhancer activity. Moreover, it is likely that some of the 27000 Treg cell-type specific H3K4me3 non-promoter regions that were identified in this study might be important for Treg cell-type specific patterns of gene expression.

Although the promoter region represents a primary element of gene expression, it is controlled by distal regulatory elements like enhancers and silencers. Previous studies have shown that H3K4me1 at promoter-distal sites was often associated with enhancer function [7], [11], [32]. Our results indicated that most of the H3K4me1 islands were cell-type specific, suggesting that enhancers are the most variable class of transcriptional regulatory element between Treg and aTconv cells, and are probably of primary importance in driving Treg cell-type specific patterns of gene expression. Our study identified a number of putative regulatory elements for genes that are highly important for Treg cell functions. For instance, we found that there was a region located in intron 1 of the FOXP3 gene (ChrX:49001620–49002192) enriched by both H3K4me1 and H3K4me3 in Treg cells and which showed enhancer activity in transfected Jurkat cells. A region located upstream of IL2RA (Chr10:6148000–6148784) also showed enhancer activity. Since cultured and expanded conventional T cells express high levels of CD25 as a consequence of TCR activation, it is possible that this region may contribute to regulating constitutive (rather than activation-induced) CD25 expression in Treg cells.

In addition, we found that most H3K4me1 enriched regions were not enriched by H3K4me3, suggesting that most potential regulatory elements were only enriched by H3K4me1 but lacked H3K4me3 in the whole genome of human Treg and aTconv cells. This finding is consistent with the observations of p300-associated enhancers that were found to have little or no H3K4me3 [32]. However, there were also some regions simultaneously enriched by H3K4me1 and H3K4me3, such as the region located in intron 1 of the FOXP3 gene, which did show enhancer activity. Whether or not the regions enriched by the two types of histone methylations may harbor more potential to act as enhancers remains unknown.

In conclusion, we identified genome-wide H3K4me1 and H3K4me3 modification regions in Treg and aTconv cells. The H3K4me3 modifications located in proximal-promoter regions were nearly identical in both Treg and aTconv cells, with the exception of a few promoters of genes, such as FOXP3 and CCR7, which are expressed uniquely in Treg cells. In contrast to the H3K4me3 modification, H3K4me1 exhibited cell-type specific locations, indicating that enhancers are the most variable class of transcriptional regulatory elements between Treg and aTconv cells. Furthermore, enhancers are likely to be of primary importance in driving Treg cell-type specific patterns of gene expression. The Treg- and aTconv-specific H3K4me1 and H3K43 patterns may function as significant mediators of differentiation events, lineage commitment and cell type-specific gene expression. It is likely that this basic principle is not confined to these two closely related T cell populations, but may apply generally to somatic cell lineages in adult organisms.

## Supporting Information

Figure S1 **Real-time PCR analysis of known target sites enriched by H3K4me3.** ChIP assays were performed with Tregs and aTconv cells as described. DNA fragments binding to H3K4me3 histones were immunoprecipitated using antibodies directed against H3K4me3. The rabbit isotype immunoglobulin G (IgG) served as control, IgGa was a control for Treg and IgGb was a control for aTconv. Precipitated DNA was quantified by real-time PCR with primers specific for the sites of the known cell signature genes and common genes. Sample PCR products were set in relation to input DNA. *, *P*<0.001 vs IgG control; #, *P*<0.001 vs aTconv.
(EPS)

Figure S2 **Real-time PCR analysis of known target sites after sequencing.** ChIP assays were performed with Tregs and aTconv cells as described. DNA fragments binding to H3K4me1 histones were immunoprecipitated using antibodies directed against H3K4me1. The rabbit isotype immunoglobulin G (IgG) served as control, IgGa was a control for Treg and IgGb was a control for aTconv. Precipitated DNA was quantified by real-time PCR with primers specific for the sites of the known cell signature genes and common genes. Sample PCR products were set in relation to input DNA. *, *P*<0.001 vs IgG control.
(EPS)

Figure S3 **H3K4me3 and H3K4me1modifications of signature genes and common genes in Treg and aTconv T cells.** Shown are the following tracks (from top to bottom): Genes location; ChIP-seq tag counts for H3K4me3 or H3K4mel modifications in Treg cells; ChIP-seq tag counts for H3K4me3 or H3K4mel modifications in aTconv cells; UCSC Genes Based on Refseq; mammalian Consensus. Red frames represent the H3K4me3 modifications in proximal promoters. Blue frames represent the Treg cell-type specific H3K4me1 enriched regions and orange frames represent the H3K4me1 regions enriched in both Treg and aTconv cells. Panel A, H3K4me3 modification on IL2RA (Chr10:6095000–6145000) loci. Panel B, H3K4me3 modification on CTLA4 (Chr2:204440000–204448000) loci. Panel C, H3K4me3 modification on GITR, i.e., TNFRSF18 (Chr1:1128000–1132500) loci. Panel D-I, H3K4me3 modification on STAT1 (Chr2:191540000–191590000), STAT2 (Chr12:55020000–55045000), STAT3 (Chr17:37720000–37800000), STAT4 (Chr2:191600000–191750000), STAT5A (Chr17:37690000–37720000) and STAT6 (Chr12:55775000–55795000) loci. Panel J H3K4me3 modification on CTLA4 (Chr2:204440000–204448000) loci. Pane K H3K4me3 modification on IL2RA (Chr10:6115000–6155000) loci. PaneL H3K4me3 modification on GITR (Chr1:1125000–1136000) loci.
(TIF)

Table S1 **Real-time PCR primers for mRNA expression of known genes.**
(DOC)

Table S2 **Primers for the amplification of H3K4me1 or H3k4me3 enriched regions.**
(DOC)

Table S3 **Summary data for ChIP-seq regions enriched in H3K4me1 or H3K4me3.**
(DOC)

Table S4 **Real-time PCR primers for the promoters of known genes.**
(DOC)

Table S5 **Real-time PCR primers for H3K4me1 sites.**
(DOC)
