# Supplementary figures and images for: Global Mapping of H3K4me1 and H3K4me3 Reveals the Chromatin State-Based Cell Type-Specific Gene Regulation in Human Treg Cells
Source: PLoS One. 2011 Nov 23;6(11):e27770. doi: 10.1371/journal.pone.0027770 (PMC3223197; doi:10.1371/journal.pone.0027770)

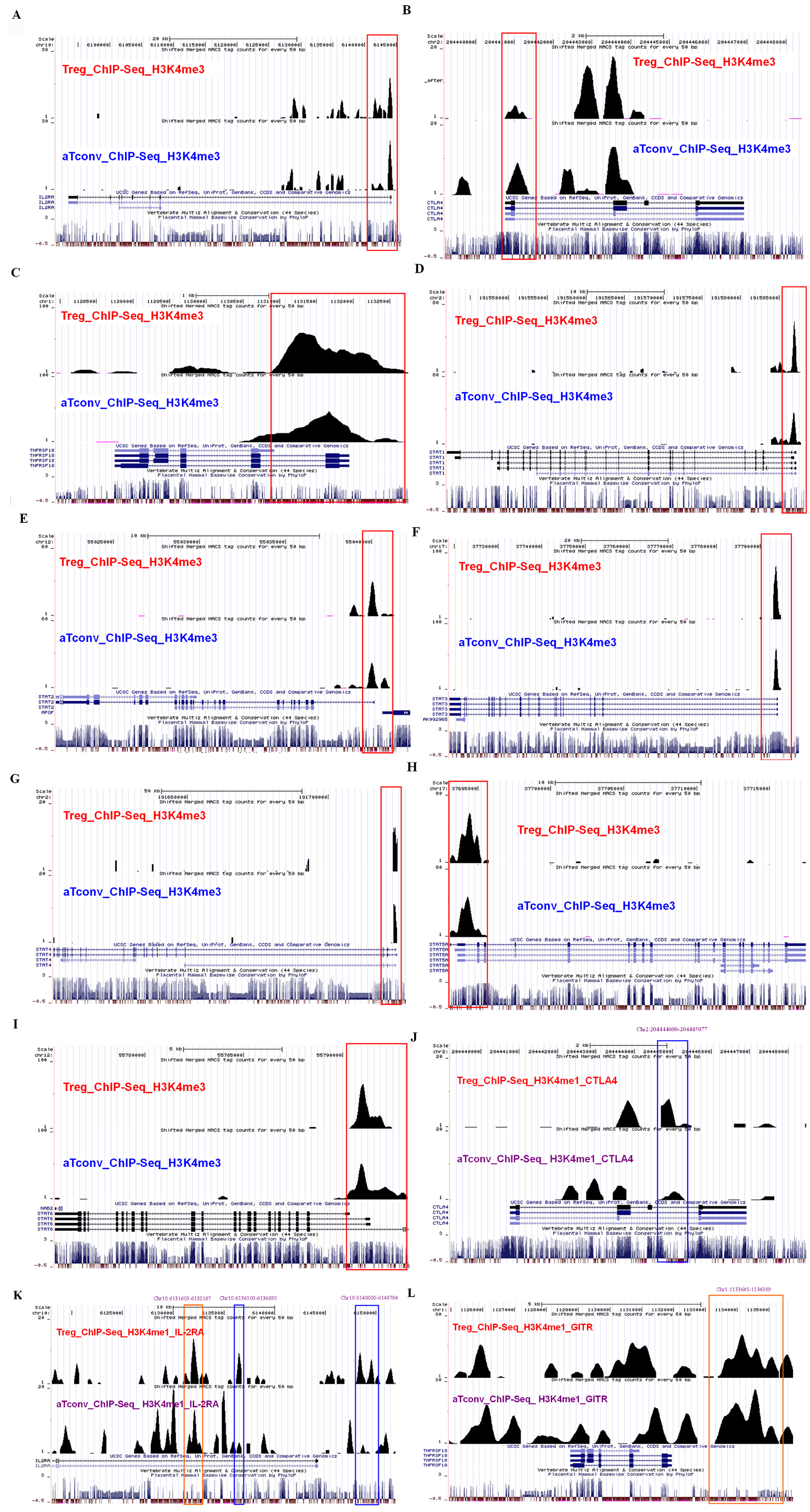

Supplement: Figure S3 — H3K4me3 and H3K4me1modifications of signature genes and common genes in Treg and aTconv T cells. Shown are the following tracks (from top to bottom): Genes location; ChIP-seq tag counts for H3K4me3 or H3K4mel modifications in Treg cells; ChIP-seq tag counts for H3K4me3 or H3K4mel modifications in aTconv cells; UCSC Genes Based on Refseq; mammalian Consensus. Red frames represent the H3K4me3 modifications in proximal promoters. Blue frames represent the Treg cell-type specific H3K4me1 enriched regions and orange frames represent the H3K4me1 regions enriched in both Treg and aTconv cells. Panel A, H3K4me3 modification on IL2RA (Chr10:6095000–6145000) loci. Panel B, H3K4me3 modification on CTLA4 (Chr2:204440000–204448000) loci. Panel C, H3K4me3 modification on GITR, i.e., TNFRSF18 (Chr1:1128000–1132500) loci. Panel D-I, H3K4me3 modification on STAT1 (Chr2:191540000–191590000), STAT2 (Chr12:55020000–55045000), STAT3 (Chr17:37720000–37800000), STAT4 (Chr2:191600000–191750000), STAT5A (Chr17:37690000–37720000) and STAT6 (Chr12:55775000–55795000) loci. Panel J H3K4me3 modification on CTLA4 (Chr2:204440000–204448000) loci. Pane K H3K4me3 modification on IL2RA (Chr10:6115000–6155000) loci. PaneL H3K4me3 modification on GITR (Chr1:1125000–1136000) loci. (TIF) [file pone.0027770.s003.tif]
