# Supplementary material for: Global Mapping of H3K4me1 and H3K4me3 Reveals the Chromatin State-Based Cell Type-Specific Gene Regulation in Human Treg Cells
Source: PLoS One. 2011 Nov 23;6(11):e27770. doi: 10.1371/journal.pone.0027770 (PMC3223197; doi:10.1371/journal.pone.0027770)
Supplement: Table S1 — Real-time PCR primers for mRNA expression of known genes. (DOC) [file pone.0027770.s004.doc]

**Table S1 Real-time PCR primers for mRNA expression of known genes**

| **Gene** | **Sense primer** | **Antisense primer** |
| --- | --- | --- |
| **FOXP3** | CATCCGCCACAACCTGA | CGTCCATCCTCCTTTCCT |
| **GITR** | GCCATTCAAGAACTCAA | CAAACTTAGCCATACAGG |
| **CTLA4** | ATTCTGACTTCCTCCTCTGG | TCATTCTGGCTCTGTTGG |
| **IL2RA** | ATTGGCTGGATGTGGT | TCAAGGACTGCTGGTATG |
| **STAT1** | GTGATTCCCTCCTGCTA | ACCCTCATTCTCGTCCT |
| **STAT2** | CAGACCAAGGACCTGTATC | AGGAGTAGGAAGGGCAAGA |
| **STAT3** | GCGATGCTTCCCTGATTG | TTGGTGGAGGAGAACTGC |
| **STAT4** | CGTTGGATTGATGGGTATG | TTGTAGTCTCGCAGGATGT |
| **STAT5** | ATAACACCCGCAACGAG | CTGCCAACACTGAACTGAGAC |
| **STAT6** | CCTCGTCACCAGTTGCTT | TCCAGTGCTTTCTGCTCC |
| **CCR7** | GCTACCTACCTGCTCAACCTG | AAGTGGACACCGAAGACCC |
