# Supplementary material for: Global Mapping of H3K4me1 and H3K4me3 Reveals the Chromatin State-Based Cell Type-Specific Gene Regulation in Human Treg Cells
Source: PLoS One. 2011 Nov 23;6(11):e27770. doi: 10.1371/journal.pone.0027770 (PMC3223197; doi:10.1371/journal.pone.0027770)
Supplement: Table S3 — Summary data for ChIP-seq regions enriched in H3K4me1 or H3K4me3. (DOC) [file pone.0027770.s006.doc]

**Table S3** Summary data for ChIP-seq regions enriched in H3K4me1 or H3K4me3

| A. ChIP-seq regions enriched in H3K4me1 | | |
| --- | --- | --- |
|  | Cell | |
|  | Treg cells Conventional CD4^+^ T Cells | |
| ChIP Ab to H3K4me1 Ab to H3K4me1 | | |
| Reads | | |
| Sequenced  Mapped  Unique mapped | 14418323 | 14741137 |
|  | 1755328 | 2404792 |
|  | 1430103 | 1932747 |
| Peaks | | |
| Counts | 49921 | 65470 |
| Average of length(bp) | 163 | 211 |
| Median of length(bp) | 168 | 221 |
| Total regionlength(bp) | 8186157 | 13857353 |
| Coverage | 0.30% | 0.51% |
| B. ChIP-seq regions enriched in H3K4me3 | | |
|  | Cell | |
|  | Treg cells Conventional CD4^+^ T Cells | |
| ChIP Ab to H3K4me3 Ab to H3K4me3 | | |
| Reads | | |
| Sequenced  Mapped  Unique mapped | 20133067 | 15380003 |
|  | 3048362 | 2280340 |
|  | 2732515 | 2048553 |
| Peaks | | |
| Counts | 50281 | 33815 |
| Average of length(bp) | 301 | 393 |
| Median of length(bp) | 555 | 355 |
| Total regionlength(bp) | 15134881 | 13293332 |
| Coverage | 0.56% | 0.44% |

Note: Percent coverages were calculated for reference genome sequence lengths of 3.080 Gb for human. Ab, antibody.
