# Supplementary material for: Global Mapping of H3K4me1 and H3K4me3 Reveals the Chromatin State-Based Cell Type-Specific Gene Regulation in Human Treg Cells
Source: PLoS One. 2011 Nov 23;6(11):e27770. doi: 10.1371/journal.pone.0027770 (PMC3223197; doi:10.1371/journal.pone.0027770)
Supplement: Table S4 — Real-time PCR primers for the promoters of known genes. (DOC) [file pone.0027770.s007.doc]

**Table S4 Real-time PCR primers for the** promoters of known genes

| **Gene promoter** | **Sense primer** | **Antisense primer** |
| --- | --- | --- |
| **FOXP3** | ATAGGTCTGCGGCTTCCA | CCTGCCATCTCCTCCAAT |
| **GITR** | GGGTTTCAAGAGCCCACAGC | GGGTCGGGATTCTCAGGTCA |
| **CTLA4** | CGGCTTCCTTTCTCGTA | AGCGGTGTTCAGGTCTT |
| **IL2RA** | CTCTTGGCAGTGGTCTCA | TTCAGTTCGCCGCATC |
| **STAT1** | GCTAAACCCAGGGAACG | GGAGGTGGAGGCAATGTA |
| **STAT2** | GGCAATCGTTTCATCTTTC | GTTATGTTGGTTCGGTATCTG |
| **STAT3** | CTGGCTTGACGGGTTGAT | CTGGTCGTGGGTAGGCTTTA |
| **STAT4** | CGTCCTCTTCCCTCTTATCC | CTACCCATCCCTTCCATC |
| **STAT5** | CCCGACAACCACATTCC | CTGCCTGCGATAGACCA |
| **STAT6** | CAGCACGAGGAGGGTTA | CAGGGAAAGGAGGTGAAGG |
