# Supplementary material for: Global Mapping of H3K4me1 and H3K4me3 Reveals the Chromatin State-Based Cell Type-Specific Gene Regulation in Human Treg Cells
Source: PLoS One. 2011 Nov 23;6(11):e27770. doi: 10.1371/journal.pone.0027770 (PMC3223197; doi:10.1371/journal.pone.0027770)
Supplement: Table S5 — Real-time PCR primers for H3K4me1 sites. (DOC) [file pone.0027770.s008.doc]

**Table S5 Real-time PCR primers** for H3K4me1 sites

| **Gene** | **Chromosomal Location (hg18)** | **Sense** | **Antisense** |
| --- | --- | --- | --- |
| **FOXP3** | ChrX:48994400-48995097 | GCAGGCAAGACAGTGGAAA | GGAAAGGAGGATGGACGAA |
| **FOXP3** | ChrX:49004128-49005080 | GGTCTACGGTCCCAAAGT | GCCCTATGAAGCAGCAA |
| **FOXP3** | ChrX:49001620-49002192 | AGGCTGCGTAGACAATAGG | TGCTTTGACCAGAGGAGTG |
| **IL2RA** | Chr10:6131603-6132187 | GTCTTTACACGGCACTCACC | AACACCCTCCTCCCTTACTG |
| **IL2RA** | Chr10:6136100-6136695 | AATGTGGAGGTAGGGAAAC | GTGTGCCAAGCAAATGAGT |
| **IL2RA** | Chr10:6148000-6148784 | GAGAAGCTAAGGGCAGAA | GTCCAGGCATTACCAACT |
| **GTIR** | Chr1:1133645-1134389 | GCACAGATGGCACAGGAGTT | TGACGGCTATGGGAAAGG |
| **CTLA4** | Chr2:204444600-204445077 | ACCTATCCCTGTTATCCA | ATTCTACCTCTTCTACCACC |
